# Scenarios Approach to the Electromagnetic Exposure: The Case Study of a Train Compartment

**DOI:** 10.1155/2015/869895

**Published:** 2015-02-23

**Authors:** A. Paffi, F. Apollonio, R. Pinto, M. Liberti

**Affiliations:** ^1^Department of Information Engineering, Electronics and Telecommunications, Sapienza University of Rome, 00184 Rome, Italy; ^2^Italian Inter-University Center of Electromagnetic Fields and Biosystems (ICEmB), 16145 Genoa, Italy; ^3^UT-BIORAD, ENEA, Centro Ricerche Casaccia, 00123 Roma, Italy

## Abstract

Previous studies identified the train compartment as the place where people can experience the highest exposure levels (still below the international guideline limits) to electromagnetic fields in the radiofrequency range. Here a possible scenario of a train compartment has been reproduced and characterized, both numerically and experimentally. A good agreement between the simulated electric field distributions and measurements has been found. Results indicate that the higher values of exposure in specific positions inside the train compartment depend on the number of active cell phones, the bad coverage condition, the cell orientation, and the presence of metallic walls. This study shows that the proposed approach, based on the scenarios characterization, may efficiently support the assessment of the individual electromagnetic exposure.

## 1. Introduction

The huge diffusion of communication technologies based on radiofrequency (RF) electromagnetic (EM) fields, such as mobile communications (GSM, UMTS) and wireless data transfer (Wi-Fi, Wi-Max, Bluetooth, ZigBee, etc.), and their massive use in crowded environments, where people last for long time periods, as schools, hospitals, offices, and transportation means, have led to concern on possible health effects of this kind of low-level multiple exposure.

As a consequence, a lot of* in vitro*,* in vivo*, and epidemiological studies have been carried out, often leading to conflicting results, as evident from literature reviews [1–6]. Literature results are even difficult to be interpreted, since there is no evidence for an assessed interaction mechanism able to explain low-level RF effects, as evidenced in recent reviews [7, 8].

A possible cause of these experimental discrepancies is an inadequate dosimetry, so clear guidelines for achieving accurate exposure conditions were proposed for both* in vitro* [9, 10] and* in vivo* [11–13] experimental studies.

For what concerns the epidemiological studies, a recent paper on a possible positive correlation between some kinds of brain cancer and cell phone exposure [14] has heightened the debate on the metric used to assess the individual level of exposure [15, 16].

An accurate individual exposure assessment is of fundamental importance not only for quantifying the exposure during the epidemiological studies, but also for choosing the dose levels when designing* in vitro* and* in vivo* experiments. The importance of the exposure assessment in the wider context of the public health assessment is evidenced by several recent studies [17–19] and by a study campaign just concluded, aiming at the evaluation of the impact of different EM sources on the individual exposure in the framework of the COST Action BM0704 [20].

A common methodology, mostly used in the exposure assessment for the epidemiological studies, is based on the employment of the exposimeters [21–24]. Exposimeters allow an objective estimation and the possibility to make an analysis time dependent and band selective. These features are very useful when dealing with long-time exposures to different EM sources. However, disadvantages of such an approach are the heavy postprocessing, the underestimation due to the body screening, and the difficulty in deriving internal EM quantities useful in planning* in vivo* and* in vitro* laboratory activities [21, 22].

A complementary approach has been proposed in [25, 26]. It is based on the identification and the characterization, both numerical and experimental, of typical scenarios in order to estimate the electric (*E*) and magnetic (*H*) fields levels in typical or worst-case exposure conditions. Possible scenarios to be studied are hospital intensive care units, hospital management, computer labs, train stations and airports, and transportation means.

In particular, in this paper, a typical train compartment has been chosen as an interesting case study; it has been reproduced and the *E* and *H* fields inside have been evaluated both numerically and experimentally. The choice of such a scenario is related to the extensive experimental study of [21], where the train has been shown as the location where the highest mean value of exposure (still well below international guideline limits) is measured. Although different EM sources are likely to be contemporarily present inside the train, the main contribution is attributable to mobile phone handsets [21], especially those based on the GSM protocol, that emit the highest power levels [27]. In particular, the higher density of mobile phone users, the bad coverage conditions often experienced during a train journey, and the presence of reflecting metallic walls could be the causes of such higher exposure levels.

In this paper, the specific goal of the train compartment characterization is to test the aforementioned hypotheses underlying the higher exposure levels and to identify possible worst-case exposure conditions.

A more general aim is to suggest an approach based on scenarios identification, to support the assessment of individual EM exposure. The approach is based on the following steps: (i) the identification of a proper scenario on the basis of* in situ* inspection, (ii) the arrangement of such scenario in a laboratory environment to carry on experimental measurements, and (iii) the accurate modeling of the scenario to perform numerical simulations. The matching from steps (ii) and (iii) will define the goodness of the numerical model and hence will permit a rapid exploration of several different configurations through the tool of the simulations. A final campaign of measurements in real sites will guarantee the reliability of the identified scenario.

This kind of approach can usefully complement other methodologies for the individual exposure assessment, such as the use of exposimeters.

## 2. Methods

### 2.1. Scenario Setup

Before moving towards the experimental and numerical characterization, the scenario has to be chosen and reproduced both in laboratory and in computer models. This preliminary activity requires a sequence of steps:

1. identification of the scenario,
2. *in situ* inspection,
3. choice of typical configurations (mean and/or worst case),
4. reproduction of the scenario.

To reproduce the chosen scenario, in terms of size, materials, and possible positions of the EM sources, a typical compartment of high-speed Italian trains was considered, and main geometrical dimensions and arrangements have been reproduced following the description in [26]. Different configurations were examined in order to identify the conditions leading to the worst-case exposure. These configurations are described in detail in Sections 2.2 and 2.3.

### 2.2. Experimental Measurements

The experimental scenario was reproduced in the Laboratories of ENEA Casaccia, Technical Unit of Radiation Biology and Human Health (UT-BIORAD).

**Figure 1:**
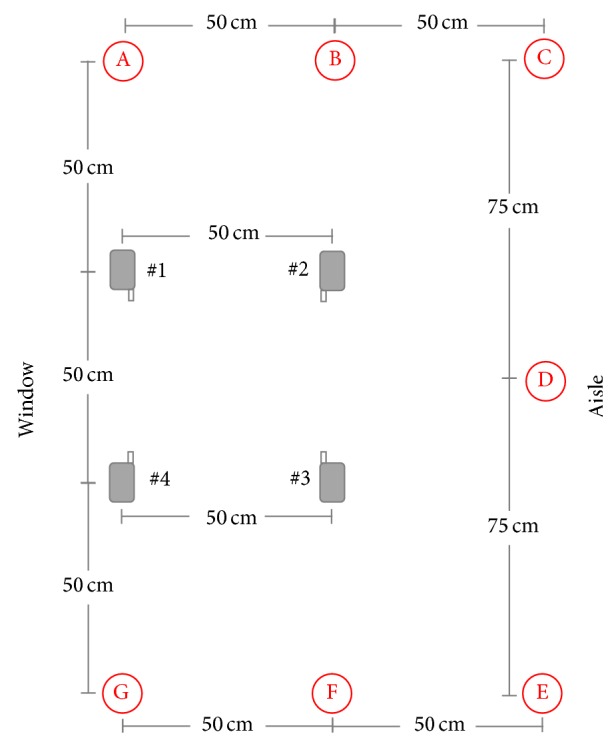
Positioning of the four cell phones (#1–#4) and of the seven measurement points (A–G) inside the train compartment scenario.

To reproduce worst-case exposure conditions, the GSM technology was chosen, transmitting higher power levels with respect to the UMTS [27]. The sources were four cell phones, GSM900 (two Motorola Timeport and two Nokia 6310), placed on a wooden table, 80 cm high, at a distance of 50 cm from each other, as in Figure 1. The cell phones, referred to as #1, #2, #3, and #4, transmitted at 900 MHz singularly or contemporarily, either at the maximum of their power (peak power: 2 W), by using controlled SIM cards, or in standard transmission conditions (average power: 250 mW). The maximum power transmission was set to simulate a bad coverage condition often experienced during a train journey.

The stability of phones emission was previously measured in a fixed point by means of a miniaturized isotropic *E* field probe (mod. ET3DV5R, Schmid & Partner Engineering AG, Zurigo, Switzerland) connected to a voltmeter (HP 3457 Hewlett Packard Corp., Palo Alto, CA, USA). During all lifetime of the battery (about two hours), the measured *E* field values presented a standard deviation *σ* < 1%, confirming the stability of emitted power.

The wide band sensor Wandel & Goltermann EMR-300 was employed to measure the root mean square (RMS) of the *E* and the *H* fields, averaged over 6 minutes (display data refresh every 4 s in “Average” modality). It was equipped with the isotropic *E* field probe Type 8.3 (100 kHz–3 GHz, measurement range 0.6–800 V/m, calibration uncertainty at 940 MHz 12%) and *H* field probe Type 10.2 (27 MHz–1 GHz, measurement range 0.025–16 A/m, calibration uncertainty at 940 MHz 12%) Wandel & Goltermann.

The sensors were mounted on a dielectric support, in the seven points (labeled from A to G) of Figure 1 at 80 and 120 cm of quote, roughly corresponding to the chest and the head of seated passengers.

With the aid of polystyrene supports, each phone was placed in different orientations (horizontal and vertical) leading to four configurations:

1. horizontal: all cell phones in horizontal orientation (see Figure 1);
2. vertical: all cell phones in vertical orientation;
3. mixed1: two facing cell phones in vertical orientation and the other two horizontal;
4. mixed2: two cell phones lying on one diagonal in vertical orientation and the other two horizontal.

Measurements were carried out in the absence and in the presence of metallic panels placed on the floor and laterally at a distance of 65 cm from the cell phones.

All measured values were reported in the text with the associated standard uncertainty coming from the measurement instrumentation specifications and the measured phones power stability. The *E* field and *H* field measurement standard uncertainties are 14.7% and 15.5%, respectively.

### 2.3. Numerical Simulations

The same train scenario was simulated using CST Microwave Studio 2010.

**Figure 2:**
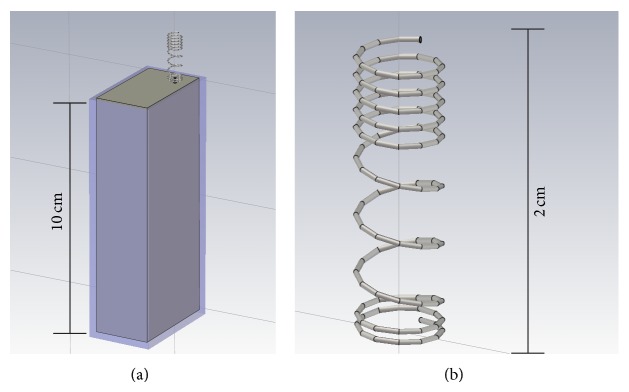
Numerical model of the cell phone (a) with a detail of the helix antenna (b).

According to [28], each cell phone was modeled as a box (0.100 × 0.043 × 0.024 m^3^) of perfect electric conductor (PEC) covered with 2 mm of plastic material (*ε*_*r*_ = 2), except for the face where a helix antenna is placed (see Figure 2). The antenna was fed using a waveguide port with 1 W input power, as defined in default conditions of CST. Suitable scaling factors, accounting for plausible values of the antenna gains, were considered in order to simulate cell phones irradiating the maximum power of 2 W [29].

The four cell phones were placed either vertically or horizontally inside an air box 1.4 × 1.7 × 1.6 m^3^. The total model was solved using the frequency solver at 900 MHz, a mesh of 50 lines per wavelength, and radiation boundary conditions.

**Figure 3:**
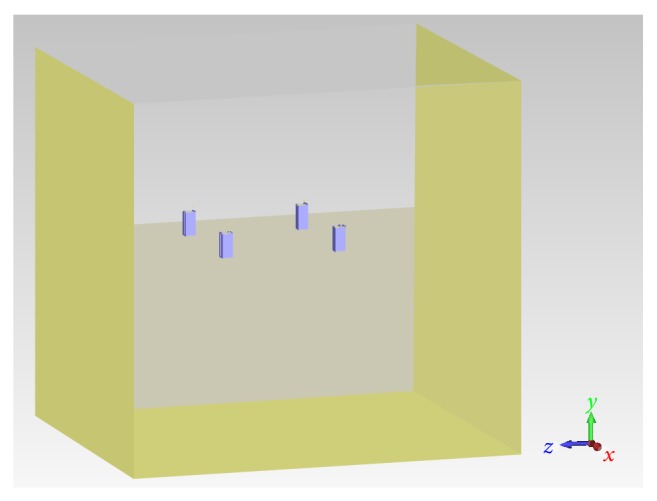
Numerical scenario with four cell phones placed in vertical orientation inside a box closed by four PEC walls: on the floor and on three sides. The wall on one side was 105 or 80 cm high to simulate the presence of a window starting at different plausible quotes.

To simulate the metallic panels, PEC walls were added at some boundaries of the simulation box, as shown in Figure 3. The panel on the side of the window was modeled with different heights (105 and 80 cm) to simulate the presence of a window at different realistic quotes.

The total *E* and *H* fields obtained by the superimposition of the fields generated by the single sources were calculated, using an* ad hoc* MATLAB procedure, by summing the field variances in each space point, under the hypothesis that the sources were statistically independent of each other.

Accounting for the integration volume of the *E* and *H* field probes, statistical values of the simulated fields, such as the mean value and the standard deviation, were calculated in a cubic box, 6 cm on a side, around each measurement point.

## 3. Results

### 3.1. Without Metallic Walls

As a preliminary step, both *E* and *H* fields were measured in order to evaluate the goodness of the far field approximation in the measurement points.  Table 1 reports the ratio |*E* | /|*H*| obtained with all cell phones in the horizontal orientation and contemporary transmitting at the maximum power; the combined uncertainty for this indirect measurement was calculated to be equal to 21.4%. Results show that the estimated ratio |*E* | /|*H*| is very close to the impedance of the free space (377 Ω) for all the measurement points. Thus, the *E* field measurements are sufficient for the experimental characterization of the scenario. The *E* and *H* field distributions closer to the sources will be obtained using numerical simulations.

As already shown in [26], in bad coverage condition, the measured *E* field in all measurement points is up to 6.2 dB higher than in standard transmission condition. Therefore, all the following results will refer to the bad coverage condition.

In order to find the worst case, the *E* field values measured when all the passengers were using the mobile phone were compared to the case of a single user.

The *E* field values were measured in correspondence of the points B, C, and D at 80 cm of quote for each cell phone (#1, #2, #3, and #4) placed in front of the B measurement point, and for all the cell phones contemporarily transmitting. In this measurement session, the cell phones were placed in the horizontal configuration.

Table 2 shows the measured values averaged over the cell phones of the same brand (#1 and #2, in the second column, #3 and #4, in the third column); the reported standard uncertainty accounts for the variability among cell phones belonging to the same group. As evident from Table 2, the phones #1 and #2 generate an *E* field lower and more variable than the #3 and #4 ones; the total field generated by the all phones contemporarily transmitting is up to 10 dB above the values obtained with the single phones.

In the following, all cell phones contemporarily transmitting at the maximum power have been considered as the worst case.

**Figure 4:**
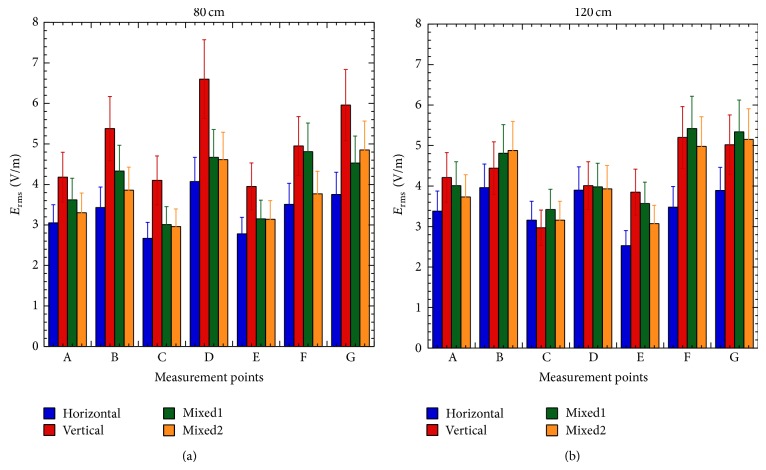
Measured *E* fields on the plane at 80 cm (a) and 120 cm (b) with the cell phones in horizontal (blue), vertical (red), and mixed (green and orange) configurations.

The other condition possibly affecting the individual exposure is the cell phones orientation.  Figure 4 reports the *E* field values measured in the four configurations described in Section 2.2 and referred to as horizontal, vertical, mixed1, and mixed2. Interestingly, at 80 cm of quote, the vertical configuration always induces the highest field values and the horizontal the lowest ones, and the mixed1 and mixed2 give similar and intermediate values. This is no longer true at 120 cm of quote. In this plane, except for the points E, F, and G, where the horizontal phones generate the lowest exposure, all configurations become almost indistinguishable.

It is also possible to note that, at 80 cm of quote, the *E* field is the lowest in C and E, that is, the farthest points from the sources, and higher in correspondence of the cell phones and between them. The *E* field tends to become more uniform as the quote increases, for example, at 120 cm.

**Figure 5:**
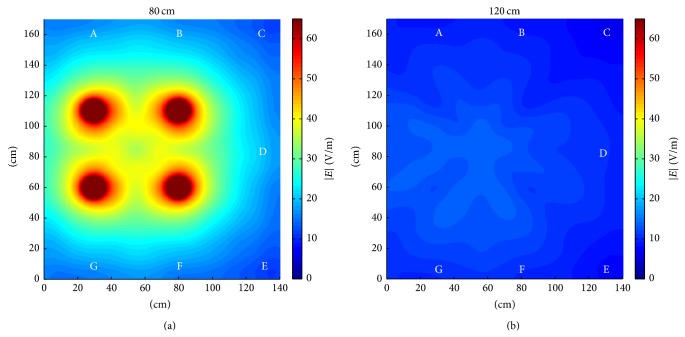
Simulated |*E*| fields generated by the four cell phones in vertical configuration on the plane at 80 cm (a) and 120 cm (b). The positions corresponding to the measurement points are evidenced by the letters A–G.

This behavior is even more evident considering the |*E*| field distributions on the planes *y* = 80 cm (Figure 5(a)) and *y* = 120 cm (Figure 5(b)) coming from numerical simulations with the cell phones in the vertical configuration.

However, it should be noticed that the numerical values refer to the *E* field amplitude continuous wave (CW). Therefore, they cannot be quantitatively compared, in a direct way, to the experimental measurements that concern the RMS *E* field generated by 900 MHz GSM sources with a maximum radiated power of 2 W, according to the GSM standard [29].

**Figure 6:**
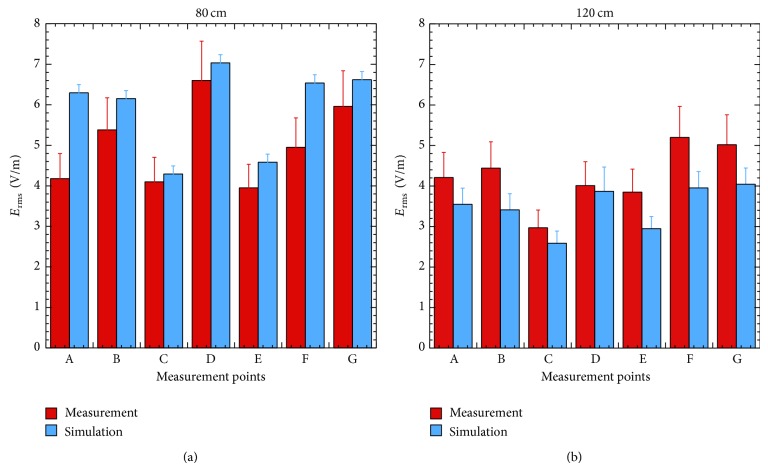
Comparison between measured and scaled simulated *E* fields on the plane at 80 cm (a) and 120 cm (b) with the cell phones in the vertical configuration.

To roughly compare numerical and experimental results, the first ones have been rescaled by a factor 4 (measured values are root mean squared and mean power of a GSM signal is 1/8 of the corresponding CW); then the obtained values have been averaged over the integration volume, as described in Section 2.3 and reported in Figure 6 together with measurements.

From simulation results of Figure 6(a) it is clear that in front of the cell phones the *E* field assumes very similar levels, whereas minimum values are present in correspondence of the farthest points (C and E) from the sources and a maximum is located in D, that is, between two active cell phones. At 120 cm (Figure 6(b)) the *E* field tends to decrease and to become more uniform (see also Figure 5) and the values in all points tend to approach those in C and E. Similar behaviors are present in the measured *E* fields even though they are not so clear due to the significant differences among actual cell phones in terms of kind of antennas and thus the actual radiated power (see Table 2 for the emitted *E* fields) and its spatial distribution.

Despite these differences among antennas, not accounted for in simulations, experimental and numerical results are almost always inside the error bars and show the same behavior in the explored domain. Such agreement allows us to use simulations to easily and quickly obtain useful support in identifying worst-case conditions.

### 3.2. With Metallic Walls

As a consequence of what is discussed at the end of Section 3.1, the effect of metallic walls in the train scenario was first checked using numerical simulations.

**Figure 7:**
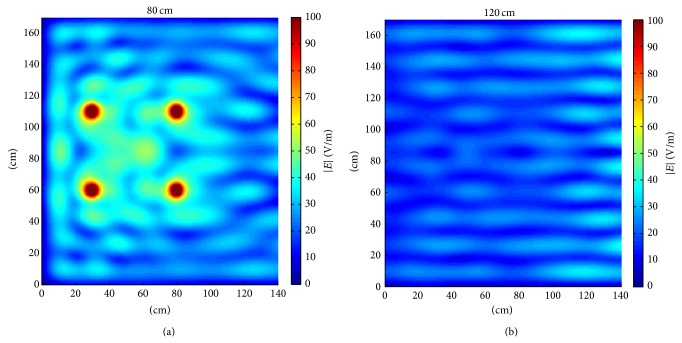
Simulated |*E*| fields generated by the four cell phones in vertical configuration on the planes at 80 cm (a) and 120 cm (b) in the presence of metallic walls with the window at 150 cm of quote.

Figure 7 shows the |*E*| field distributions at the quotes of 80 cm (panel (a)) and 120 cm (panel (b)) for the window starting at 150 cm of quote. As evident from Figure 7, the maximum *E* field values increase with respect to the case in the absence of metallic walls; this increase reaches 15 dB for the 120 cm plane in several points depending on the relative position of the cell phones with respect to the metallic walls. Indeed, Figure 7 shows standing waves due to the wave reflections at the boundaries and the consequent presence of space regions where the fields combine in a constructive way.

By lowering the window's quote of 25 cm, the *E* field distributions are very similar to those of Figure 7 (data not shown) and the average exposure inside the scenario does not significantly change (less than 1 dB). Only a decrease of about 2 dB is detected in some regions of the 80 cm plane.

**Figure 8:**
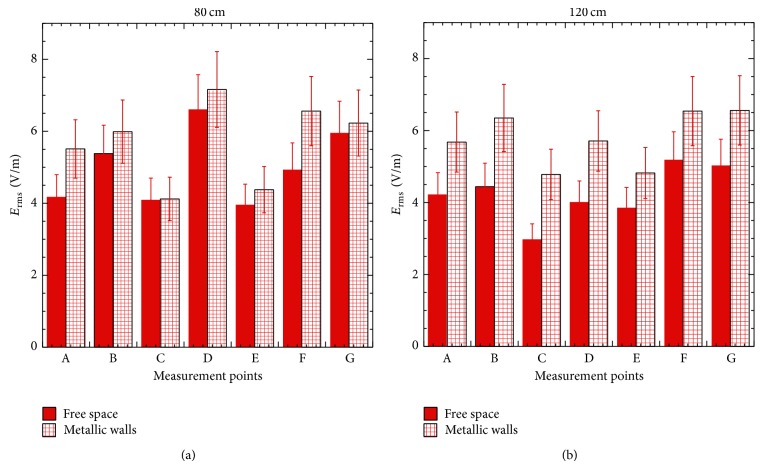
Comparison between the measured *E* fields with (gridded red) and without (full red) metallic walls on the plane at 80 cm (a) and 120 cm (b) with the cell phones in the vertical configuration.

The simulated scenario was then reproduced in the laboratory using movable metallic panels. Experimental results confirm the numerical prediction. The comparison between measured values in the presence and in the absence (free space) of metallic walls (Figure 8) shows a significant increase of the *E* field that becomes even more significant (up to 4 dB) at the quote of 120 cm.

Such results confirm the hypothesis that the presence of metallic walls is one of the causes of the high individual exposure levels inside the train together with the bad coverage conditions that maximize the power emitted.

The numerical model can be further complicated, by changing the positions of the metallic walls in the train and accounting for the presence of passengers and other metallic and dielectric objects, in order to obtain more realistic results.

## 4. Discussion and Conclusions

In this work, a scenario representing a train compartment has been numerically modeled, reproduced in laboratory, and completely characterized.

Results of this study show a good agreement between simulated and measured *E* fields at the measurement points; thus the simulated scenario has been used to identify worst-case conditions (the presence of metallic walls), which have been successively implemented in laboratory.

Numerical and experimental results confirm that while remaining below the limits imposed by the international regulations [30], the individual exposure in the train may significantly increase due to the bad coverage condition, the high number of cell phones which are contemporarily transmitting, and the presence of metallic walls. In particular the increases of the *E* field caused by the bad coverage, the four cell phones contemporarily transmitting, and the presence of the metallic walls are in the order of 6, 10, and 4 dB, respectively.

The effect of the cell phone orientation is also important (vertical cell phones generate higher exposure levels) but only in the points closer to the sources, that is, on the 80 cm plane.

The case study of the train compartment, independently of the particular transmitting technology chosen for the EM sources, confirms the possibility of using the scenario characterization to integrate other methodologies in the individual exposure assessment.

**Figure 9:**
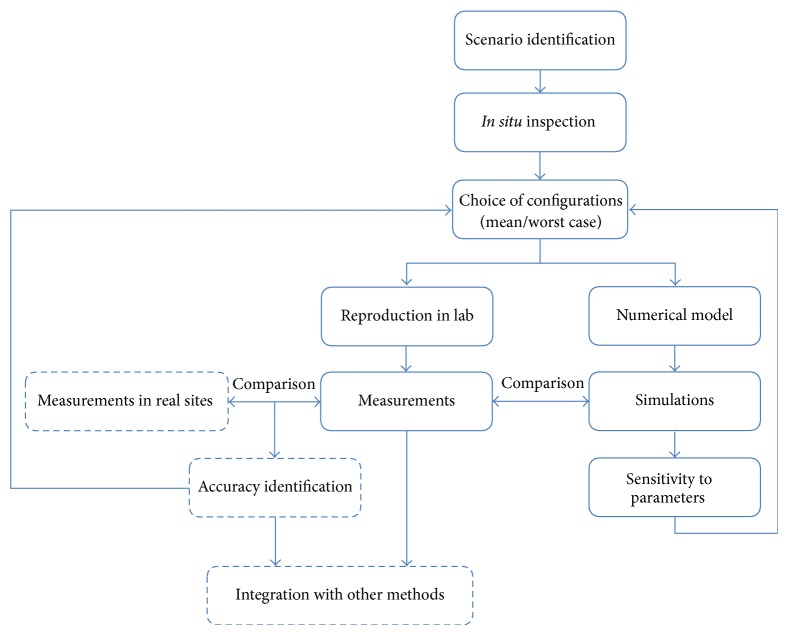
Block diagram describing the proposed approach for the assessment of the individual EM exposure inside a scenario.

The approach we want to suggest is completely described in the block diagram of Figure 9.

The blocks contoured by solid lines summarize all the steps described in this paper, where measurement and simulations are integrated to choose the most interesting scenario and exposure conditions. The subsequent steps are represented in Figure 9 by dashed blocks. They consist of a measurement campaign in real sites to identify the accuracy of the measurements carried out in the reproduced scenario, that is, how much the obtained characterization is reliable and representative of the exposure conditions in actual sites. This latter step may lead to changes in the chosen configuration. Finally, the obtained data have to be integrated into those coming from other methodologies, such as the exposimeters, to obtain the best assessment of the EM exposure.

## Figures and Tables

**Table 1:** Ratio between the *E* and *H* fields measured in all points at the two different quotes: 80 and 120 cm.

Measurement points	|*E*|/|*H*| (Ω)	
	80 cm	120 cm
A	369 ± 79	389 ± 83
B	387 ± 83	386 ± 83
C	398 ± 85	309 ± 66
D	375 ± 80	385 ± 82
E	372 ± 80	389 ± 83
F	366 ± 78	387 ± 83
G	384 ± 82	380 ± 81

**Table 2:** *E* field values measured in points B, C, and D at 80 cm of quote when only one source (#1, #2, #3, and #4), placed in front of the B measurement point, or all sources were transmitting. The cell phones are in the horizontal configuration.

Measurement points	*E*_rms_ (V/m)		
	#1 or #2	#3 or #4	All
B	0.96 ± 0.39	1.44 ± 0.21	3.05 ± 0.45
C	0.67 ± 0.50	1.26 ± 0.19	3.43 ± 0.50
D	1.06 ± 0.42	1.59 ± 0.23	4.54 ± 0.67

## References

[1] Repacholi M. H. (1998). Low-level exposure to radiofrequency electromagnetic fields: Health effects and research needs. *Bioelectromagnetics*.

[2] Ahlbom A., Green A., Kheifets L., Savitz D., Swerdlow A. (2004). Epidemiology of health effects of radiofrequency exposure. *Environmental Health Perspectives*.

[3] Vecchia P., Matthes R., Ziegelberger G., Lin J., Saunders R., Swerdlow A. (2009). *Exposure to High Frequency Electromagnetic Fields, Biological Effects and Health Consequences (100 kHz–300 GHz)*.

[4] Röösli M., Frei P., Mohler E., Hug K. (2010). Systematic review on the health effects of exposure to radiofrequency electromagnetic fields from mobile phone base stations. *Bulletin of the World Health Organization*.

[5] Advisory Group on Non-Ionising Radiation (2012). Health effects from radiofrequency electromagnetic fields (RCE-20).

[6] Repacholi M. H., Lerchl A., Röösli M. (2012). Systematic review of wireless phone use and brain cancer and other head tumors. *Bioelectromagnetics*.

[7] Sheppard A. R., Swicord M. L., Balzano Q. (2008). Quantitative evaluations of mechanisms of radiofrequency interactions with biological molecules and processes. *Health Physics*.

[8] Apollonio F., Liberti M., Paffi A. (2013). Feasibility for microwaves energy to affect biological systems via nonthermal mechanisms: a systematic approach. *IEEE Transactions on Microwave Theory and Techniques*.

[9] Kuster N., Schönborn F. (2000). Recommended minimal requirements and development guidelines for exposure setups of bio-experiments addressing the health risk concern of wireless communications. *Bioelectromagnetics*.

[10] Paffi A., Apollonio F., Lovisolo G. A. (2010). Considerations for developing an RF exposure system: a review for in vitro biological experiments. *IEEE Transactions on Microwave Theory and Techniques*.

[11] Kuster N., Torres V. B., Nikoloski N., Frauscher M., Kainz W. (2006). Methodology of detailed dosimetry and treatment of uncertainty and variations for in vivo studies. *Bioelectromagnetics*.

[12] Paffi A., Apollonio F., Lovisolo G. A., Marino C., Liberti M. Exposure systems for bioelectromagnetic investigations in the radiofrequency range: classification and emerging trends.

[13] Paffi A., Merla C., Pinto R. (2013). Microwave exposure systems for in vivo biological experiments: a systematic review. *IEEE Transactions on Microwave Theory and Techniques*.

[14] Cardis E., Armstrong B. K., Bowman J. D. (2011). Risk of brain tumours in relation to estimated RF dose from mobile phones: rsults from five interphone countries. *Occupational and Environmental Medicine*.

[15] Bürgi A., Frei P., Theis G. (2010). A model for radiofrequency electromagnetic field predictions at outdoor and indoor locations in the context of epidemiological research. *Bioelectromagnetics*.

[16] Frei P., Mohler E., Bürgi A. (2010). Classification of personal exposure to radio frequency electromagnetic fields (RF-EMF) for epidemiological research: evaluation of different exposure assessment methods. *Environment International*.

[17] Kelsh M. A., Shum M., Sheppard A. R. (2011). Measured radiofrequency exposure during various mobile-phone use scenarios. *Journal of Exposure Science and Environmental Epidemiology*.

[18] Aerts S., Deschrijver D., Joseph W. (2013). Exposure assessment of mobile phone base station radiation in an outdoor environment using sequential surrogate modeling. *Bioelectromagnetics*.

[19] Gajšek P., Ravazzani P., Wiart J., Grellier J., Samaras T., Thuróczy G. (2013). Electromagnetic field exposure assessment in Europe radiofrequency fields (10 MHz-6 GHz). *Journal of Exposure Science and Environmental Epidemiology*.

[20] http://www.cost.eu/domains_actions/bmbs/Actions/BM0704.

[21] Frei P., Mohler E., Neubauer G. (2009). Temporal and spatial variability of personal exposure to radio frequency electromagnetic fields. *Environmental Research*.

[22] Neubauer G., Cecil S., Giczi W. (2010). The association between exposure determined by radio frequency personal exposimeters and human exposure: a simulation study. *Bioelectromagnetics*.

[23] Lauer O., Neubauer G., Röösli M. (2012). Measurement setup and protocol for characterizing and testing radio frequency personal exposure meters. *Bioelectromagnetics*.

[24] Thielens A., De Clercq H., Agneessens S. (2013). Personal distributed exposimeter for radio frequency exposure assessment in real environments. *Bioelectromagnetics*.

[25] Liberti M., Apollonio F., Paffi A. Experimental characterization of possible scenarios for the individual exposure assessment.

[26] Paffi A., Apollonio F., Colotti R. Characterization of a train compartment scenario for the individual exposure assessment.

[27] Nitu V., Lojewski G. (2014). Comparison of the average output power of GSM and UMTS mobile phones and the impact in exposure to electromagnetic waves. *UPB Scientific Bulletin C*.

[28] Pisa S., Cavagnaro M., Lopresto V., Piuzzi E., Lovisolo G. A., Bernardi P. (2005). A procedure to develop realistic numerical models of cellular phones for an accurate evaluation of SAR distribution in the human head. *IEEE Transactions on Microwave Theory and Techniques*.

[29] European Telecommunications Standards Institute (ETSI) (1996). GSM technical specifications.

[30] ICNIRP (1998). Guidelines for limiting exposure to time-varying electric, magnetic, and electromagnetic fields (up to 300 GHz). *Health Physics*.

